# A fatal case of concurrent EBV-induced meningitis, ARDS, and HLH in an immunocompetent adult: a case report

**DOI:** 10.1186/s12985-026-03281-4

**Published:** 2026-08-19

**Authors:** Jie Tian, Jingjun Dong, Wei Guan

**Affiliations:** Department of Respiratory and Critical Medicine, Affiliated Hospital Group of Guangdong Medical University, Shenzhen Baoan Central Hospital (Baoan Central Hospital of Shenzhen), Shenzhen, China

**Keywords:** Epstein-Barr virus, Hemophagocytic lymphohistiocytosis, Meningitis, Acute Respiratory Distress Syndrome, Fulminant course.

## Abstract

**Rationale:**

Epstein-Barr virus (EBV) typically causes self-limited infections in immunocompetent adults. Life-threatening complications such as hemophagocytic lymphohistiocytosis (HLH), meningitis, and acute respiratory distress syndrome (ARDS) are exceptionally rare and have not been previously reported to occur concurrently in a single patient. This case highlights the catastrophic potential of EBV and underscores a critical therapeutic dilemma: balancing the need for aggressive immunosuppressive therapy to control life‑threatening HLH against the risk of exacerbating uncontrolled EBV viremia and severe infections in a critically ill patient.

**Patient concerns:**

A 34-year-old immunocompetent woman presented with a 10-day history of high-grade fever (39.9 °C) and persistent headache.

**Diagnoses:**

The diagnosis of Epstein-Barr virus (EBV)-associated hemophagocytic lymphohistiocytosis (HLH) was confirmed by meeting 5 of 8 HLH-2004 criteria in the setting of extreme EBV viremia (1.48 × 10⁸copies/mL), with high EBV DNA load additionally detected by metagenomic next-generation sequencing (mNGS) in cerebrospinal fluid (239 EBV reads) and lower respiratory tract samples (145,386 EBV reads). Concurrently, EBV meningitis was confirmed by CSF findings (lymphocytic pleocytosis, elevated protein, and hypoglycorrhachia) alongside positive mNGS. She also met the Berlin criteria for ARDS, with bilateral pulmonary infiltrates on imaging and a PaO₂/FiO₂ ratio of 200 prior to ICU admission (on low-flow oxygen, consistent with moderate ARDS). Shortly after ICU admission, her oxygenation deteriorated to a PaO₂/FiO₂ ratio of 81 on mechanical ventilation with PEEP of 10 cmH₂O, meeting criteria for severe ARDS.

**Interventions:**

Management was restricted to organ support (mechanical ventilation, continuous renal replacement therapy), ganciclovir antiviral therapy, and broad-spectrum antibiotics. Crucially, guideline-directed immunomodulatory therapy for HLH (e.g., corticosteroids/etoposide per HLH-2004) was not administered due to delayed diagnosis, lack of institutional expertise, and socioeconomic constraints.

**Outcomes:**

The patient’s condition deteriorated rapidly, with progression to fatal multi-organ failure and death on hospital day 5 (illness day 15).

**Lessons:**

This case demonstrates that EBV can trigger a fulminant, lethal triad of HLH, meningitis, and ARDS even in immunocompetent hosts, which has not been previously reported in a single patient. It underscores that high-risk EBV-HLH mandates immediate immunomodulation, as antiviral and supportive care alone are catastrophically insufficient. Early recognition and overcoming barriers to administering complex, urgent therapies are essential to improving outcomes.

## Introduction

Epstein-Barr virus (EBV), a ubiquitous gamma-herpesvirus, that infects more than 95% of adults worldwide, with most primary infections occurring during childhood or adolescence and seroconversion completed by early adulthood [1]. While immunocompetent individuals typically experience asymptomatic infection or self-limited infectious mononucleosis (IM), life-threatening complications—including hemophagocytic lymphohistiocytosis (HLH), malignant lymphoma, and liver failure—occur predominantly in immunocompromised hosts [2–4]. Although EBV is the most commonly recognised trigger of secondary HLH, documented cases of EBV-induced encephalitis or meningitis are relatively uncommon in adults [5–6]. Furthermore, while infection with several respiratory viruses, notably SARS-CoV-2 and influenza, is an established cause of acute respiratory distress syndrome (ARDS), EBV-associated ARDS is extremely rare [7–8]. Crucially, however, no published cases describe the concurrent emergence of these three EBV-driven complications—HLH, meningitis, and ARDS—in a single patient. We report a novel case of fulminant primary EBV infection in an immunocompetent woman manifesting this lethal triad. The infection culminated in fatal multi-organ failure within 15 days of symptom onset. This rare constellation of complications underscores EBV’s catastrophic potential in immunologically intact hosts and offers important lessons for clinical practice.

## Case presentation

A 34‑year‑old immunocompetent woman, previously fit and well, with no family history of note. She presented with a 10-day history of high-grade fever (peaking at 39.9 °C), headache, chills, rigors, nausea, vomiting, myalgia, fatigue, and anorexia, without respiratory symptoms. On examination, she reported no rash, lymphadenopathy, joint pain, coryzal symptoms, eye redness, personality change, reduced consciousness, or visual disturbance. Initial chest CT (Fig. 1) revealed bilateral miliary nodules predominantly in the upper lobes, along with scattered small patchy consolidations, prompting admission for suspected pulmonary infection. On admission, vital signs were stable; auscultation was clear, but neurological examination revealed nuchal rigidity with negative Kernig’s and Brudzinski’s signs. Physical examination revealed splenomegaly on abdominal palpation, confirmed by bedside ultrasound; no hepatomegaly was detected. Initial laboratory findings on admission showed mild leukopenia (WBC 3.18 × 10⁹/L, normal 4–10 × 10⁹/L), with neutrophil count (1.98 × 10⁹/L, normal 1.8–7.2 × 10⁹/L) and lymphocyte count (1.09 × 10⁹/L, normal 0.8–4.0 × 10⁹/L) both at the lower limits of normal; hemoglobin was 113 g/L (normal 115–150 g/L) and platelet count was 126 × 10⁹/L (normal 100–300 × 10⁹/L). By hospital day 2, however, the blood counts had declined significantly, with WBC 1.43 × 10⁹/L, hemoglobin 87 g/L, and platelets 82 × 10⁹/L, indicating the development of pancytopenia. These cytopenias continued to worsen over the following days (Table 1). Elevated CRP 9.6 mg/L (normal < 6 mg/L), hyponatremia (Na⁺ 125 mmol/L, normal 135–145 mmol/L), hypochloremia (Cl⁻ 94 mmol/L, normal 98–107 mmol/L), hypocalcemia (Ca²⁺ 1.99 mmol/L, normal 2.1–2.6 mmol/L), significant hepatic dysfunction (Total bilirubin 33.1 µmol/L, normal 5.1–19 µmol/L; Direct bilirubin 21.7 µmol/L, normal 0–10 µmol/L; ALT 678 U/L, normal 0–40 U/L; AST 697 U/L, normal 0–40 U/L; GGT 473 U/L, normal 0–45 U/L; ALP 633 U/L, normal 50–130 U/L; albumin 27.4 g/L, normal 35–50 g/L), and coagulopathy (APTT 47.0 s, normal 24–36 s; TT 22.6 s, normal 11–18 s; FIB 1.5 g/L, normal 2–4 g/L; D-dimer 3.22 mg/L, normal < 0.55 mg/L). Arterial blood gas (FiO₂ 21%) showed mild hypoxemia (PaO₂ 71 mmHg, PaCO₂ 31 mmHg, pH 7.45, OI 338, lactate 1.3 mmol/L). Lumbar puncture yielded clear cerebrospinal fluid (CSF) with elevated pressure (190 mmH₂O, normal 70–180 mmH₂O), positive Pandy’s test(elevated globulins), mild pleocytosis (WBC 6 × 10⁶/L, 90% mononuclear), elevated protein (0.54 g/L, normal 0.15–0.45 g/L), and hypoglycorrhachia (glucose 2.10 mmol/L vs. serum 4.3 mmol/L). CSF metagenomic next-generation sequencing (mNGS) detected Epstein-Barr virus (239 reads), confirming EBV meningitis; quantitative PCR for EBV in CSF was not performed due to limited sample volume, and mNGS was prioritized as it offers higher sensitivity for pathogen detection in CSF. CSF autoantibody testing (anti-MOG, anti-NMDA, anti-AQP4) was not performed as the CSF findings and positive EBV mNGS were consistent with EBV meningitis, making autoimmune encephalitis less likely. Extensive infectious workup was performed. Blood cultures (aerobic and anaerobic) and urine cultures were negative. A respiratory viral panel (including influenza A/B, RSV, adenovirus, parainfluenza, and human metapneumovirus) was negative. Serological testing for HIV, syphilis, HBV, HCV, CMV, HSV‑1/2, VZV, and toxoplasma was negative or showed past infection without active disease. Lymphocyte subset analysis performed on day 2 showed normal T-cell counts (CD3⁺ 1012 cells/µL, CD4⁺ 571 cells/µL, CD8⁺ 412 cells/µL; normal ranges 955–2860, 550–1440, and 320–1250 cells/µL, respectively). Follow-up analysis on day 4 revealed marked declines in T-cell subsets (CD3⁺ 483 cells/µL, CD4⁺ 236 cells/µL, CD8⁺ 205 cells/µL), as well as NK cells (13.64 cells/µL) and NKT cells (24.75 cells/µL).

**Table 1:** Serial Laboratory Parameters During Hospitalization

Parameter (Unit)	Day 1	Day 2	Day 3		Day 4	Day 5		Reference Range
**Hematology**								
WBC (×10⁹/L)	3.18	1.43		2.10	2.22	3.86	4–10	
Neutrophils (×10⁹/L)	1.98	0.96		1.48	1.66	1.39	1.8–7.2	
Hemoglobin (g/L)	113	87		78	94	42	110–160	
Platelets (×10⁹/L)	126	82		67	44	37	100–300	
Lymphocytes (%)	34.20	30.40		29.20	24.90	61.50	20–40	
Lymphocytes (×10⁹/L)	1.09	0.43		0.61	0.55	2.37	0.8-4.0	
**Inflammation**								
CRP (mg/L)	9.6	15.8		18.1	51.5	55.4	0–6	
IL-6(pg/mL)		37.02			1621.54		0–7	
Procalcitonin (ng/mL)	0.02	0.26		0.36	13.55	5.93	0-0.05	
**HLH-related markers**								
Ferritin (µg/L)		> 2000					15–150	
Triglycerides (mmol/L)	4.79	3.53		3.24	3.50	3.25	0-1.71	
**Liver function**								
Total Bilirubin (µmol/L)	33.1	39.9		61.0	91.4	106.4	5.1–19.0	
Direct Bilirubin (µmol/L)	21.7	33.1		48.6	74.4	88.6	0–10	
Indirect Bilirubin (µmol/L)	11.4	6.8		12.4	17.0	17.8	0–15	
Albumin (g/L)	27.4	20.0		28.7	30.1	21.7	35–50	
ALT (U/L)	678	647		445	413	302	0–40	
AST (U/L)	697	832		592	900	2454	0–40	
GGT (U/L)	473	337		277	229	87	0–40	
ALP (U/L)	633	604		518	399	230	50–130	
**Coagulation**								
PT (s)	13.2	18.6		16.1	20.6	39.4	11–16	
APTT (s)	47.0	64.1		56.3	99.4	> 200	24–36	
PTA (%)	83.8	40.1		65.1	36.3	15.9	75–120	
TT (s)	22.6	28.6		20.7	24.9	67.2	11–18	
Fibrinogen (g/L)	1.5	0.9		1.6	1.2	< 0.30	2–4	
D-Dimer (mg/L)	3.22	23.52		13.81	38.22	37.96	0-0.55	
**Renal function**								
BUN (mmol/L)	2.9	-		3.0	5.1	6.5	2.5–7.1	
Creatinine (µmol/L)	51	-		46	77	230	45–84	
**Cardiac markers**								
Troponin I (ng/mL)	0.02	0.21		0.156	0.317	10.709	0.1–0.2	
Myoglobin (ng/mL)	20.11	18.21		13.57	501.2	4820.58	2-68.9	
LDH (U/L)	813	981		1082	2334	6096	120–250	
CK (U/L)	18	29		42	516	4442	26–140	
NT-proBNP (pg/ml)	23.45	302.43		486.31	1021.46	5972.62	0-125	
**Pancreatic enzymes**								
Amylase (U/L)	-	-		-	64	224	28–100	
Lipase (U/L)	-	-		-	59	697	13–60	
**Blood gas**								
PaO₂ (mmHg)	71	58		81	82	39	80–100	
PaCO₂ (mmHg)	31	29		33	33	33	35–45	
pH	7.45	7.43		7.42	7.41	6.95	7.35–7.45	
Lactate (mmol/L)	1.3	2.8		1.3	3.2	> 15	0.5–1.6	
Oxygenation Index (PaO₂/FiO₂)	338	200		81	126	98	> 400	
**Virological studies**								
Whole blood mNGS (EBV reads)	-	-		-	122,765	-	Negative	
LRT mNGS (EBV reads)	-	-		-	145,386	-	Negative	
Plasma EBV-DNA (copies/mL)	-	-		-	-	1.48 × 10^8^	< 500	
**Infectious Disease Screening**								
Human Immunodeficiency Virus	Negative						Negative	
Syphilis	Negative						Negative	
Hepatitis B Virus	Negative						Negative	
Hepatitis C Virus	Negative						Negative	
**Autoimmune Screening**								
ANA		Negative					Negative	
Ds-DNA		Negative					Negative	
ESR(mm/h)		70					0–20	
**Immunological parameters**								
CD45^+^CD3^+^ T cells (cells/uL)		1012			483	-	955–2860	
CD3^+^CD4^+^ T cell (cells/uL)		571			236		550–1440	
CD3^+^CD8^+^ T cell (cells/uL)		412			205		320–1250	
NK cells (cells/uL)	-	-			13.64	-	120–814	
NKT cells (cells/uL)	-	-			24.75	-	20–349	
IgG (g/L)	-	-		17.61	-	-	7–16	
IgA (g/L)	-	-		2.29	-	-	0.7-4.0	
IgM (g/L)				0.95			0.4–2.3	
C3 (g/L)				1.59			0.9–1.8	
C4 (g/L)				0.57			0.1–0.4	
**Cerebrospinal fluid**								
Opening Pressure (mmH₂O)	190	-		-	-	-	70–180	
Appearance	Clear	-		-	-	-	Clear	
Pandy’s Test	Positive	-		-	-	-	Negative	
RBC (×10⁶/L)	1000	-		-	-	-	0	
WBC (×10⁶/L)	6	-		-	-	-	0–5	
Mononuclear Cells (%)	90	-		-	-	-	< 30	
Glucose (mmol/L)	2.10	-		-	-	-	2.2–3.9	
(Simultaneous Blood Glucose)	4.3	-		-	-	-	3.9–6.1	
Protein (g/L)	0.54	-		-	-	-	0.15–0.45	
LDH (U/L)	23	-		-	-	-	< 40	
Chloride (mmol/L)	118	-		-	-	-	120–130	
ADA (U/L)	0.8	-		-	-	-	0–9	
mNGS (EBV reads)	-	-		239	-	-	Negative	

Comprehensive hematologic, inflammatory, hepatic, coagulation, renal, cardiac, metabolic, and immunologic profiles from admission (Day 1) to terminal stage (Day 5) in a patient with fulminant EBV-associated HLH, meningitis, and ARDS. Critical deterioration is evidenced by extreme cytopenias, coagulopathy (notably profound hypofibrinogenemia), escalating hepatic injury, hyperferritinemia, hypertriglyceridemia, and metabolic acidosis. Virologic confirmation of disseminated EBV infection via mNGS and quantitative PCR is included. Reference ranges provided for context. Abbreviations: LRT = lower respiratory tract; mNGS = metagenomic next-generation sequencing

**Fig. 1:**
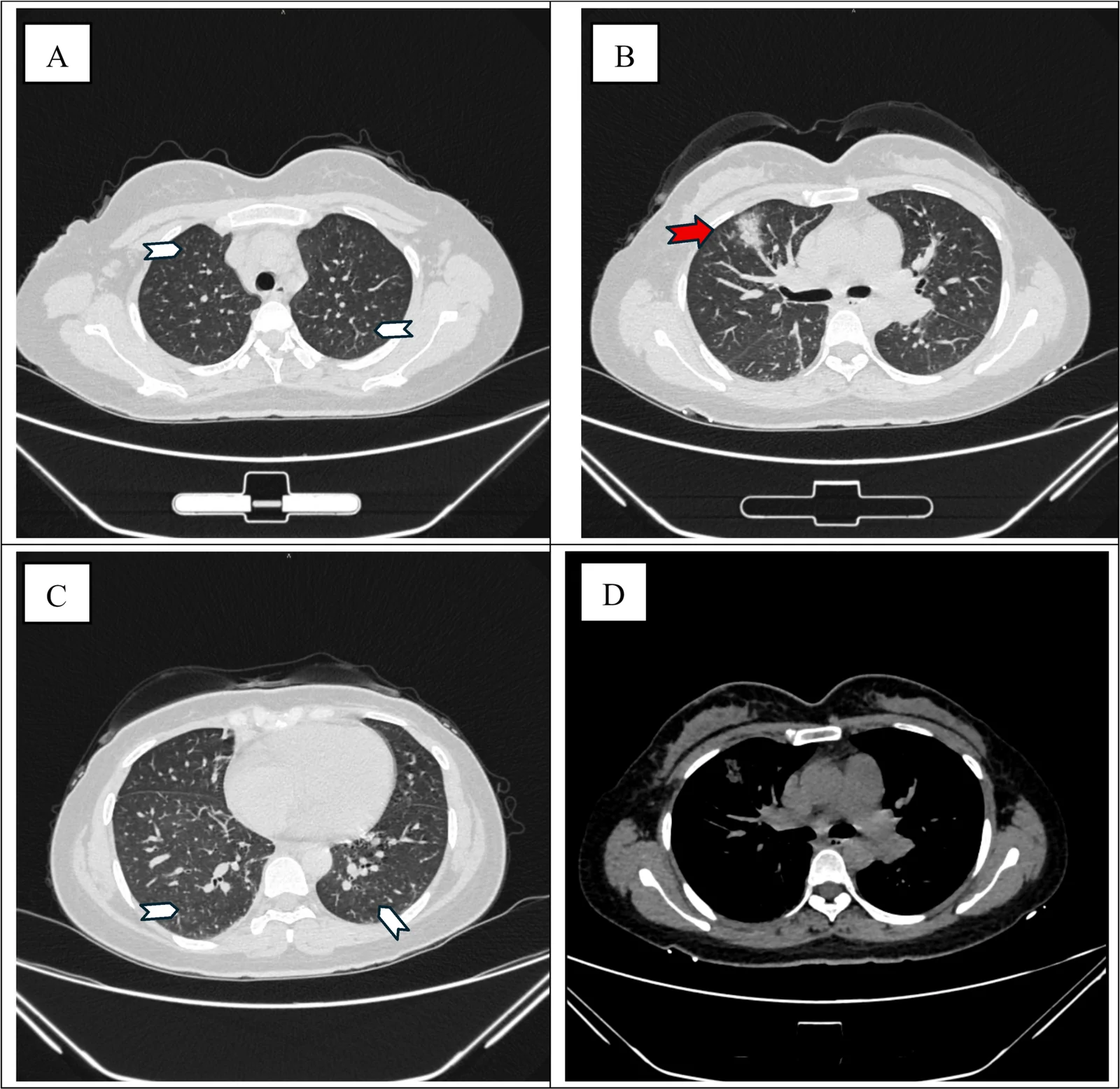
Initial chest CT imaging at admission. (**A**) Axial CT image (lung window) at the level of the lung apices. White arrowheads highlight the characteristic miliary nodular opacities scattered throughout both upper lobes. (**B**) Axial CT image (lung window) at the subcarinal level. Red arrowheads indicate the presence of small, patchy consolidations amidst the background of military nodules. (**C**) Axial CT image (lung window) at the level of the lung bases. The military nodules persist in both lower lobes, though to a lesser extent than the upper lobes, demonstrating the basilar relative sparing. (**D**) Axial CT image (mediastinal window) at the subcarinal level. This view confirms the absence of significant lymphadenopathy

Screening for malignancy and autoimmune disorders was negative; abdominal CT scan confirmed splenomegaly (Fig. 2), with morphometric measurements revealing a spleen length of 14 cm, thickness of 11 cm, and width of 6 cm, all of which are significantly above normal limits. Empirical levofloxacin was initiated alongside supportive care. On hospital day 2 (illness day 12), her condition deteriorated precipitously, manifesting shock (BP 69/41 mmHg), progressive hypoxemic respiratory failure (SpO₂ 88% on low-flow O₂ via nasal cannula; arterial blood gas on FiO₂ 29%: PaO₂ 58 mmHg, PaCO₂ 29 mmHg, pH 7.43, lactate 2.8 mmol/L, PaO₂/FiO₂ ratio 200), and worsening multi-organ dysfunction. Laboratory tests revealed profound cytopenias (WBC 1.43 × 10⁹/L, Plt 82 × 10⁹/L, Hb 87 g/L), escalating hepatic failure (TBil 39.9 µmol/L, AST 832 U/L), hypertriglyceridemia (TG 3.53 mmol/L), hyperferritinemia (> 2000 µg/L), and severe coagulopathy (D-dimer 23.52 mg/L, FIB 0.9 g/L, APTT 64.1 s, PTA 40.1%). A comprehensive overview of the serial laboratory parameters throughout hospitalization is provided in Table 1. Chest X-ray demonstrated diffuse bilateral infiltrates (Fig. 3). Combined with the acute onset, hypoxemia (PaO₂/FiO₂ ratio 200 < 300), and bilateral radiographic opacities not fully explained by cardiac failure, she met the Berlin criteria for moderate ARDS. CSF metagenomic next-generation sequencing (mNGS) detected Epstein-Barr virus (EBV) (239 reads), confirming EBV meningitis. Concurrently, she met 5 of 8 HLH-2004 diagnostic criteria (Body temperature > 38.5 degrees Celsius for more than 7 days, Tmax 39.5℃, splenomegaly, cytopenias, hypertriglyceridemia, hyperferritinemia), fulfilling diagnostic criteria for HLH. In addition, the HScore—a validated diagnostic tool for adult HLH—was calculated as 224, corresponding to a 96–98% probability, further supporting the diagnosis. A bone marrow aspirate was not performed to confirm hemophagocytosis, as the patient deteriorated rapidly and developed refractory respiratory and circulatory failure before the procedure could be undertaken. A liver biopsy was also not performed for the same reason. The absence of histopathological confirmation has been acknowledged as a limitation. Immediate transfer to the ICU ensued. Despite intubation and mechanical ventilation for worsening respiratory failure, vasopressor support for refractory shock, and initiation of ganciclovir, multi-organ failure progressed rapidly over the next 48 h. This included disseminated intravascular coagulation (DIC) with profound coagulopathy (PT 39.4 s, APTT > 200 s, PTA 15.9%, TT 67.2 s, FIB < 0.30 g/L, D-dimer 37.96 mg/L), hepatic failure (TBil 106.4 µmol/L, DBil 88.6 µmol/L, Albumin 21.7 g/L, ALT 302 U/L, AST 2454 U/L), acute kidney injury (serum creatinine 230 µmol/L) requiring continuous renal replacement therapy (CRRT), myocardial injury (Troponin I 10.709 ng/mL, Myoglobin 4820.58 µg/L), pancreatitis (LPS 697 U/L, AMYL 224 U/L), and gastrointestinal hemorrhage evidenced by coffee-ground emesis and positive occult blood testing. Subsequent mNGS testing confirmed high-load EBV viremia (whole blood: 122,765 reads; lower respiratory tract sample: 145,386 reads; cerebrospinal fluid:239 reads) with quantitative EBV DNA reaching 1.48 × 10⁸ copies/mL, indicating disseminated EBV infection involving blood, lungs, and central nervous system (CNS), and confirming EBV as the underlying cause of the HLH. Despite maximal supportive care, plasma exchange, CRRT, and broad-spectrum antibacterial therapy (meropenem, levofloxacin) combined with antiviral therapy (ganciclovir), the patient succumbed to irreversible multi-organ failure on hospital day 5 (illness day 15).

**Fig. 2:**
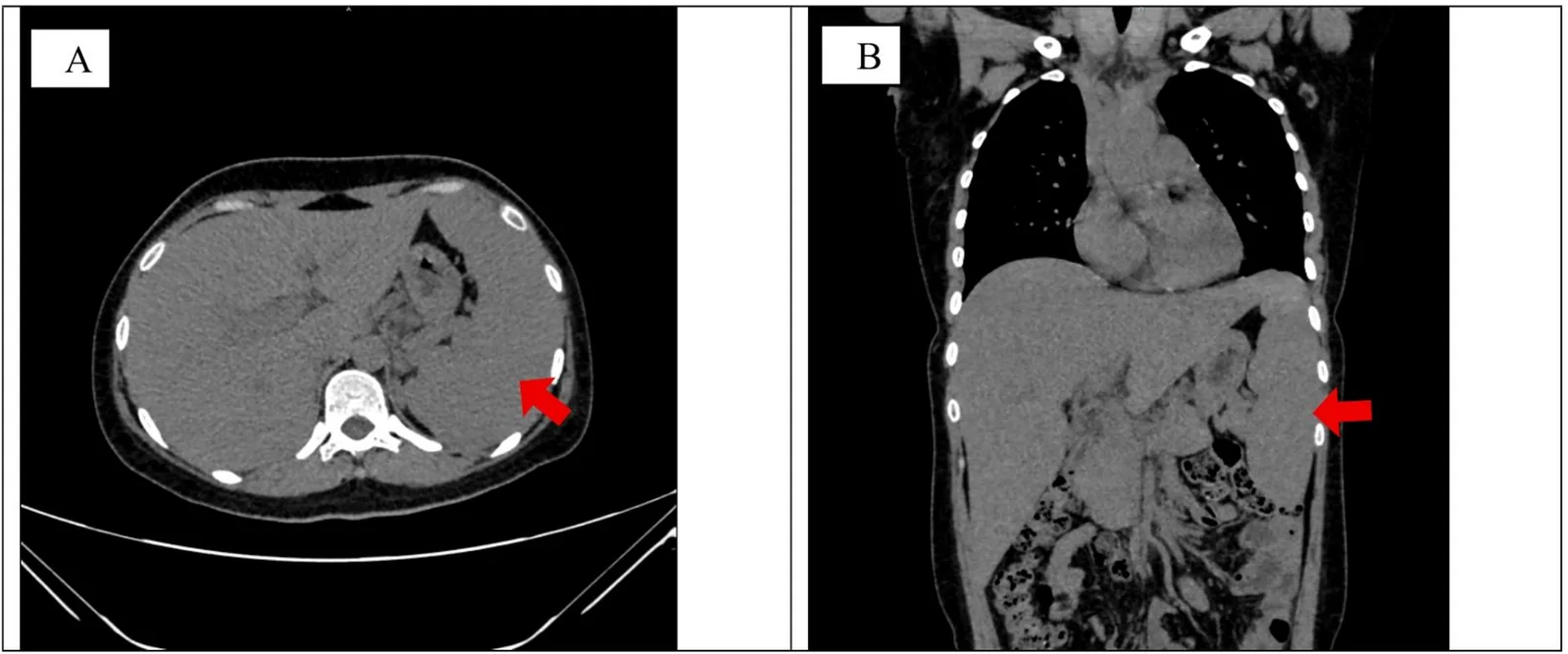
Abdominal CT demonstrating splenomegaly. (**A**) Axial CT image at the level of the splenic vein demonstrates an markedly increased splenic thickness, measuring 11 cm (normal < 5 cm). (**B**) Coronal reformatted CT image shows an enlarged spleen, with a craniocaudal length of 13 cm (normal < 12 cm)

**Fig. 3:**
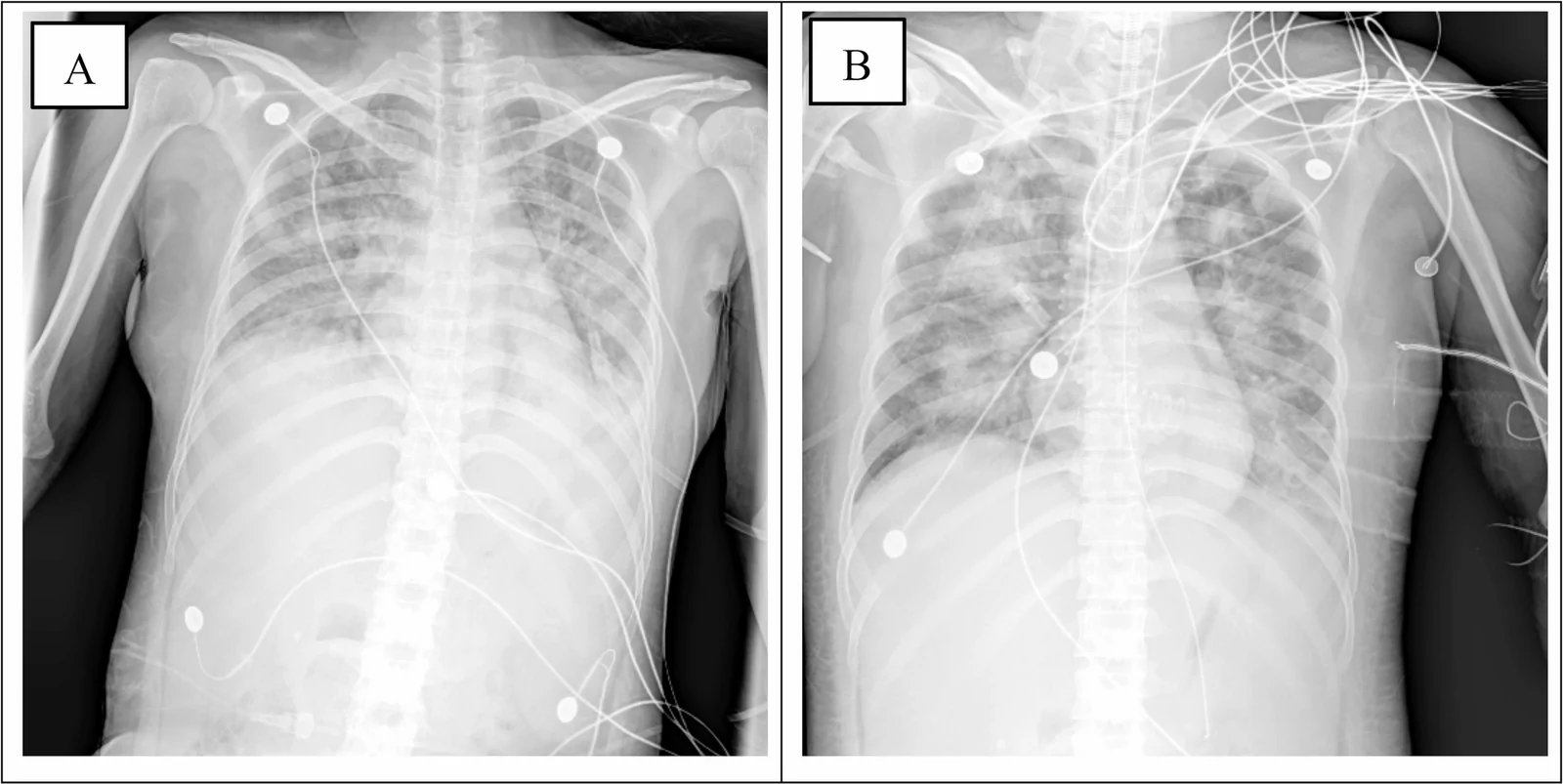
Serial chest radiographs demonstrating rapid progression to ARDS. (**A**) Anteroposterior portable chest radiograph obtained immediately prior to ICU transfer. Diffuse bilateral air-space opacities are evident, consistent with extensive pulmonary infiltration. (**B**) Anteroposterior portable chest radiograph on ICU day 2. Interval progression of the diffuse bilateral opacities is noted, with marked worsening in the upper lung zones (arrows), indicative of rapidly worsening ARDS

## Discussion

Epstein-Barr virus (EBV), also known as human gammaherpesvirus 4, is near-ubiquitous in the human population, with over 95% of adults worldwide harboring the virus, which is primarily transmitted via saliva [1]. EBV infection manifests with highly diverse clinical presentations. EBV establishes lifelong latent infection with limited gene expression, while lytic reactivation—triggered by immediate-early proteins Zta and Rta—involves full viral genome expression and replication [9]. In our patient, the extreme EBV DNA load suggests massive lytic reactivation as the trigger for the hyperinflammatory cascade. While the majority of infected individuals remain lifelong asymptomatic carriers, symptomatic primary infection—presenting as infectious mononucleosis (IM) with fever, pharyngitis, lymphadenopathy, and fatigue—is most common in adolescents and young adults [10]. Symptomatic infection in immunocompetent adults is uncommon, and severe complications like chronic active EBV infection (CAEBV), EBV-HLH, EBV-related lymphomas, or leukemias are rare [1–2, 11]. Among these, EBV-HLH carries the gravest prognosis, often presenting with a fulminant course [12–13]. While severe EBV complications like EBV-HLH or EBV meningitis are typically associated with genetic predispositions or immunodeficiencies, our case involved an immunocompetent adult with no underlying comorbidities who developed catastrophic, ultimately fatal complications, representing an exceedingly rare occurrence.

HLH is a rare and life-threatening condition, more frequently diagnosed in children than adults [14–15]. The lack of standardized diagnostic criteria for adult HLH often leads to delayed diagnosis or misdiagnosis. Consequently, the HLH-2004 diagnostic criteria remain widely applied [15]. Our patient fulfilled 5 of the 8 HLH-2004 criteria: fever, splenomegaly, cytopenias, hypertriglyceridemia, and hyperferritinemia, establishing the diagnosis of HLH. In addition to the HLH-2004 criteria [16], the HScore is a validated diagnostic scoring system for reactive HLH in adults that incorporates nine weighted variables—including immunosuppression, fever, organomegaly, cytopenias, ferritin, triglycerides, fibrinogen, AST, and bone marrow hemophagocytosis—to estimate the probability of HLH [17]. Notably, the HScore has been validated in critically ill populations and may offer superior diagnostic accuracy in the ICU setting compared to the HLH-2004 criteria, as it accounts for immunosuppression—a common confounder—and demonstrates higher specificity [18]. In our patient, the calculated HScore was 224 (96–98% probability), corroborating the diagnosis established by the HLH-2004 criteria. However, several limitations should be acknowledged: bone marrow hemophagocytosis, a component of the score, may not be readily available in rapidly deteriorating patients, though its exclusion only marginally reduces accuracy; the score was primarily developed in non-critically ill populations, warranting further prospective ICU validation; and fulminant presentations may outpace completion of all variables. Nonetheless, the HScore remains a valuable adjunctive tool alongside the HLH-2004 criteria to enhance diagnostic confidence and facilitate timely therapeutic decisions.

EBV infection is the most commonly described trigger for secondary HLH [5]. Crucially, comprehensive investigation revealed extremely high-level EBV viremia (quantitative EBV DNA in blood: 1.48 × 10⁸ copies/mL; normal range < 5 × 10² copies/mL), with mNGS exclusively detecting EBV in blood, lower respiratory tract (LRT) samples, and cerebrospinal fluid (CSF). Screening for malignancy and autoimmune disorders was negative, and there was no family history of HLH or immunodeficiency. Primary HLH was considered unlikely based on these clinical features, although it could not be definitively excluded without genetic testing. Therefore, EBV was identified as the most likely trigger of HLH in this case.

This case involved a previously healthy, immunocompetent individual who developed EBV-HLH by day 11 of illness, culminating in rapid demise, consistent with a fulminant form of EBV-HLH. Reports of such fulminant EBV-HLH are predominantly described in East Asian populations (e.g., Japan, China) and relatively rare in Western or African cohorts, suggesting potential genetic susceptibility factors [19]. Several independent prognostic factors for poor outcomes in EBV-HLH have been identified, including high EBV viral load, hypofibrinogenemia, and elevated ALT [18–19]. High EBV DNA load correlates significantly with disease severity and mortality risk; studies indicate that patients with EBV DNA > 16,100 copies/mL exhibit lower survival rates and a higher association with T/NK-cell malignancies [20]. Our patient’s exceptionally high viral load (1.48 × 10⁸ copies/mL) was a major contributor to her poor prognosis. Furthermore, she exhibited significant hypofibrinogenemia. Research indicates that EBV-positive T/NK-cell lymphoma-associated HLH patients often present with lower fibrinogen levels, correlating with worse outcomes [20–21]. Hypofibrinogenemia (≤ 1.5 g/L) has also been identified as an independent adverse prognostic factor in EBV-HLH, significantly associated with reduced 60-day and overall survival [17]. Markedly elevated ALT (678 U/L; normal 0–40 U/L) and bilirubin were evident early in our patient’s course. In EBV-HLH, elevated ALT often accompanies other markers of hyperinflammation (e.g., high cytokine levels), signifying systemic inflammatory response and organ damage, and portends a poorer prognosis, particularly if persistent and refractory to treatment [21–22].

EBV-HLH carries a high mortality rate, especially during the initial treatment phase [23]. Early initiation of HLH-directed therapy is paramount for improving survival, particularly in patients exhibiting high-risk features like elevated EBV load, hypofibrinogenemia, and ALT elevation, which indicate intense disease activity [21, 23–24]. The cornerstone treatment for EBV-HLH centers on rapidly suppressing dysregulated immune activation, blocking cytokine storms, rescuing organ function, and eliminating triggers. The core regimen primarily involves corticosteroids combined with etoposide [25–26]. For patients with EBV-infected B cells, rituximab effectively clears infected cells and improves prognosis [5]. Hematopoietic stem cell transplantation (HSCT) represents the definitive therapeutic approach for refractory/relapsed EBV-HLH, cases with inherited immunodeficiencies, chronic active EBV infection, or EBV-infected T/NK cells [27–28]. In EBV-HLH management, the role of antiviral therapy remains ambiguous. Ganciclovir may be considered when high EBV viral loads are detected in the blood, but it is generally not recommended in the absence of elevated viral burden, as antiviral efficacy remains unestablished and its potential to exacerbate HLH-associated bone marrow suppression, which could worsen pre-existing thrombocytopenia and neutropenia [29–30]. In our case, the patient received only ganciclovir antiviral therapy without early initiation of HLH-directed treatment. This critical therapeutic delay stemmed from a variety of factors. Foremost, there was a failure in clinical recognition; the diagnoses of EBV infection and subsequent HLH were not initially considered, leading to a missed opportunity for early intervention. This diagnostic gap was compounded by institutional limitations; the absence of a dedicated hematology department and a lack of experience among the ICU and respiratory teams in managing HLH. Finally, socioeconomic constraints intervened; the patient’s financial situation and the family’s explicit decision against aggressive, costly therapies ultimately precluded the administration of guideline-directed treatment even after the diagnoses were established. Consequently, management was restricted to ganciclovir (antiviral), supportive organ care, and complication management, without implementing the HLH-2004 protocol. This omission of early HLH-2004 therapy likely contributed significantly to the devastating outcome.

EBV-associated neurological infections predominantly occur in immunocompromised patients and are exceedingly rare in immunocompetent individuals [6]. Our patient presented with fever and persistent headache (unrelieved by antipyretics), nausea, and vomiting. Neurological examination revealed nuchal rigidity but negative Kernig’s and Brudzinski’s signs. Lumbar puncture demonstrated mild pleocytosis (mononuclear predominance), elevated protein, mildly elevated opening pressure, and notably, hypoglycorrhachia (CSF glucose 2.10 mmol/L vs. serum 4.3 mmol/L). While CSF findings were otherwise suggestive of viral meningitis, the significant hypoglycorrhachia is atypical. Typical viral meningitis usually shows normal or mildly decreased CSF glucose; marked reduction is uncommon. A similar case report documented significantly low CSF glucose in EBV meningitis [31]. The mechanism underlying profound hypoglycorrhachia in EBV meningitis is likely multifactorial, involving immune-inflammatory glucose consumption, virus-mediated metabolic alterations, warranting further investigation [6, 32]. Prognosis for EBV meningitis in immunocompetent patients is usually favorable [6]. Poor outcomes are typically linked to immunodeficiency, CAEBV, underlying malignancy (lymphoma, leukemia), or concurrent HLH [29, 33–34]. In our case, the fatal outcome was directly attributable to fulminant EBV-HLH and multi-organ failure, not the EBV meningitis itself.

While several respiratory viruses (e.g., influenza, SARS-CoV-2, and adenovirus) are well-established causes of ARDS, EBV-associated ARDS is relatively rare [35–36]. Our patient developed acute, progressive dyspnea and refractory hypoxemia unresponsive to oxygen therapy. Chest imaging revealed diffuse bilateral infiltrates, and arterial blood gas analysis confirmed a PaO₂/FiO₂ ratio < 300, meeting the Berlin criteria for ARDS. Critically, mNGS of a lower respiratory tract specimen detected EBV as the sole pathogen with a very high read count (145,386 reads), strongly implicating direct EBV-induced lung injury and ARDS. To our knowledge, EBV monoinfection causing ARDS in an immunocompetent adult without comorbidities has not been previously reported. Documented cases of EBV-associated ARDS usually involve: (1) Co-infection with other respiratory viruses [37–38], or (2) ARDS secondary to the systemic inflammatory cascade and multi-organ failure in EBV-HLH [39]. The absence of other pathogens in our patient’s lower respiratory tract sample argues against co-infection, suggesting that ARDS likely stemmed from the overwhelming systemic inflammation driven by EBV-HLH. This highlights the need for vigilant monitoring of respiratory function in EBV-HLH patients for the potential development of ARDS.

Notably, our patient was initially admitted to the respiratory department with a working diagnosis of pulmonary infection, underscoring the diagnostic challenge of EBV-associated pulmonary involvement. The pulmonary manifestations of EBV are heterogeneous, ranging from direct viral infection of lung tissue (pneumonitis) to indirect injury from the systemic inflammatory response, as seen in HLH-associated ARDS. Clinically, EBV pneumonitis can be non-specific, featuring fever, cough, and dyspnea, but may also be paucisymptomatic. Radiologically, its patterns are diverse, including diffuse ground-glass opacities, consolidations, centrilobular nodules, and interstitial infiltrates [8]. In our patient, the initial CT finding of bilateral miliary nodules with upper lobe predominance and scattered patchy consolidations was highly suggestive of miliary tuberculosis (TB). This radiological similarity, combined with the concomitant meningeal signs (headache, nuchal rigidity) and CSF abnormalities (lymphocytic pleocytosis, hypoglycorrhachia), logically led to the initial strong suspicion of miliary TB and tuberculous meningitis.

Several key points can aid in differentiating EBV-associated pulmonary lesions from miliary TB. First, the disease tempo and systemic inflammatory profile are distinct. While TB often follows a more subacute course [40], our patient’s rapid progression to fulminant multi-organ failure and ARDS within days was highly atypical for TB and pointed towards a hyperinflammatory state like HLH. Second, the rapid development of profound pancytopenia, extreme hyperferritinemia, and severe coagulopathy is highly characteristic of HLH and less common in early miliary TB [41]. Third, definitive microbiological and molecular evidence is crucial. In our case, the absence of acid-fast bacilli on smear, negative TB cultures, and, most importantly, the failure to detect Mycobacterium tuberculosis in CSF, blood, or lower respiratory tract samples by mNGS effectively ruled out TB. Conversely, the exclusive and high-load detection of EBV in these compartments confirmed disseminated EBV infection as the cause of both the HLH and the pulmonary involvement. Therefore, in patients presenting with a miliary nodular pattern and neurological symptoms, while TB must be aggressively ruled out, the constellation of rapid clinical deterioration, features of HLH, and negative TB microbiological tests should prompt consideration of severe disseminated viral infections like EBV as the underlying cause of the pulmonary and systemic disease.

HLH is a notoriously underrecognized condition in critically ill patients, often masquerading as sepsis or multi‑organ dysfunction, and is consequently associated with delayed diagnosis and high mortality [42]. Early recognition requires a high index of suspicion across multiple specialties—including intensive care, infectious diseases, haematology, rheumatology, immunology, and general medicine—as the clinical manifestations frequently overlap with other common conditions in the ICU setting [43]. Our case underscores the critical need for multidisciplinary education and awareness to improve timely diagnosis and management of HLH, particularly in resource‑limited settings where diagnostic tools and therapeutic options may be constrained.

We acknowledge that several factors limited a more comprehensive etiological investigation in this case. The patient’s rapid and fulminant clinical deterioration precluded extensive diagnostic procedures, including genetic testing for primary HLH and bone marrow biopsy to confirm hemophagocytosis. Postmortem examination was also not performed, which limited our ability to fully elucidate histopathological changes in affected organs. Nevertheless, the compelling clinical presentation, the highly consistent laboratory findings, and the definitive evidence from multiple mNGS tests strongly support the diagnosis of disseminated EBV infection driving the lethal triad of HLH, meningitis, and ARDS.

## Conclusion

This report presents the first documented case of concurrent EBV-induced meningitis, ARDS, and HLH in an immunocompetent adult, highlighting EBV’s catastrophic potential. The fulminant course culminating in death within 15 days after symptom onset underscores a critical imperative: EBV-HLH with high-risk markers (hypofibrinogenemia, extreme viremia, or ALT elevation) demands immediate, aggressive immunomodulatory therapy (HLH-2004 protocol). Supportive care and antivirals alone are insufficient; therapeutic delay in such presentations carries invariably fatal consequences.

## Data Availability

No datasets were generated or analysed during the current study.
